# MicroRNA-9 Reveals Regional Diversity of Neural Progenitors along the Anterior-Posterior Axis

**DOI:** 10.1016/j.devcel.2010.11.018

**Published:** 2011-01-18

**Authors:** Boyan Bonev, Angela Pisco, Nancy Papalopulu

**Affiliations:** 1 Faculty of Life Sciences, Michael Smith Building, University of Manchester, Oxford Road, Manchester M13 9PT, UK

## Abstract

Neural progenitors self-renew and generate neurons throughout the central nervous system. Here, we uncover an unexpected regional specificity in the properties of neural progenitor cells, revealed by the function of a microRNA—miR-9. miR-9 is expressed in neural progenitors, and its knockdown results in an inhibition of neurogenesis along the anterior-posterior axis. However, the underlying mechanism differs—in the hindbrain, progenitors fail to exit the cell cycle, whereas in the forebrain they undergo apoptosis, counteracting the proliferative effect. Among several targets, we functionally identify *hairy1* as a primary target of miR-9, regulated at the mRNA level. *hairy1* mediates the effects of miR-9 on proliferation, through Fgf8 signaling in the forebrain and Wnt signaling in the hindbrain, but affects apoptosis only in the forebrain, via the p53 pathway. Our findings show a positional difference in the responsiveness of progenitors to miR-9 depletion, revealing an underlying divergence of their properties.

## Highlights

► miR-9 function in neural progenitors is context dependent ► miR-9 loss induces proliferation in the hindbrain and apoptosis in the forebrain ► The primary function of miR-9 in neurogenesis is to downregulate *hairy1* mRNA levels ► miR-9/*hairy1* affects proliferation via cyclinD/p27 and apoptosis via p53

## Introduction

During neurogenesis, proliferating neural cells (neural progenitor or neural stem cells), located in the ventricular zone (VZ), undergo self-renewal to replenish the progenitor population or, alternatively, engage in asymmetric divisions associated with the generation of neurons (Götz and Huttner, 2005). The process of neurogenesis is tightly coupled with the process of regional specification, which dictates the identity of neurons born in different areas of the central nervous system (CNS) (Gaspard and Vanderhaeghen, 2010). Neural stem cells themselves have different positional identity and can give rise to tumors with different signatures depending on their origin (Lee da et al., 2010; Palm and Schwamborn, 2010).

However, how regional specificity is integrated with the fundamental cellular decisions that drive neurogenesis is not well understood. Both intrinsic and external factors are thought to contribute to the correct execution and the transition from the transcriptional programs of neural stem cells to differentiated neurons in a region-specific manner (Falk et al., 2008; Jessell, 2000; Lee and Pfaff, 2003; Marklund et al., 2010).

MicroRNAs are a class of small noncoding RNAs, which have been shown to play key roles in many developmental processes including stem cell proliferation and differentiation (Gangaraju and Lin, 2009; Kosik, 2006; Stefani and Slack, 2008). They are particularly attractive for their potential to coordinate the response of many target genes, thereby acting as point of information integration. Knockout of the essential component of microRNA-processing Dicer has shown that microRNAs are indispensable for proper neural development in zebrafish (Giraldez et al., 2005) and mouse (De Pietri Tonelli et al., 2008), although the key miRs and their precise molecular targets have not been fully examined.

miR-9 is a highly conserved microRNA, which is expressed primarily in the CNS (Kapsimali et al., 2007; Wienholds et al., 2005). In vertebrates the function of miR-9 has been studied in fish and mice with loss and gain-of-function approaches. In the fish, miR-9 has been shown to be necessary to define the mid-hindbrain boundary (MHB), a non-neurogenic boundary zone with organizer properties (Leucht et al., 2008). However, with respect to the role of miR-9 in neuronal differentiation and proliferation, the results obtained by the loss-of-function experiments in different systems have not always been consistent. In the anterior hindbrain, where miR-9 is expressed, a decrease in neuronal differentiation was reported, which, however, was not accompanied by an increase in progenitor proliferation (Leucht et al., 2008). This is similar to the result obtained in the embryonic mammalian forebrain, where miR-9 knockdown caused a reduction of early-born Cajal-Retzius neurons but did not have an effect on progenitors (Shibata et al., 2008). In another study, miR-9 knockdown caused a reduction in neural progenitors derived from mouse ES cells, accompanied by a slight increase in GFAP^+^ astrocytes, although the effects on proliferation were not directly tested (Krichevsky et al., 2006). However, the opposite result was obtained in neural stem cells derived from adult mammalian forebrain, where miR-9 knockdown caused a small increase in proliferating cells (1.37-fold) but did not change differentiation (Zhao et al., 2009). Finally, in neural progenitors derived from human ES cells, loss of miR-9 has been shown to suppress proliferation, albeit by a small degree. In this system, loss of miR-9 promoted migration of neural progenitors (Delaloy et al., 2010). From these studies the emerging theme is that in most systems, miR-9 is necessary for neuronal differentiation, but the effect on proliferation is highly variable. Differences in the results obtained may be partly due to different model systems or experimental methodology; however, these discrepancies also raise the possibility that the function of miR-9 in neurogenesis and proliferation is highly context dependent.

Here, we have undertaken a systematic analysis of miR-9 expression and function along the anterior-posterior (AP) axis during *X*. *tropicalis* development and uncovered an unexpected regional specificity. In the forebrain, miR-9 is expressed in both neural progenitors and developing neurons, whereas in the more posterior regions of the brain (mid- and hindbrain), it is restricted to neural progenitors only. Using loss-of-function experiments, we demonstrate that even though miR-9 is required for neuronal differentiation, regardless of the position along the AP axis, it regulates neural progenitors in a region-specific manner—it limits progenitor proliferation and promotes neuronal fate throughout the neural tube; in addition, in the forebrain it is important for progenitor survival. We have identified several genes that contain miR-9 binding sites in their 3′UTR and respond to miR-9 in vitro and in vivo. However, functional analysis showed that *hairy1* is the single key target that mediates the effects of miR-9 in the forebrain and the hindbrain. *hairy1* is a member of the Hes family of genes, and we show that, unlike other Hes genes, it is primarily expressed in neurogenic rather than boundary areas of the CNS (Baek et al., 2006). Finally, we provide a molecular explanation for the regional-specific effects: miR-9 regulates proliferation by feeding into the network controlling cyclinD1/p27 expression in both areas, through Wnt signaling in the hindbrain and Fgf8 signaling in the forebrain, but affects apoptosis via the mdm2/p53 pathway specifically in the forebrain. These findings suggest that the positional embryonic origin of neural progenitors is an important parameter that dictates their response to the same microRNA and that in the case of miR-9 the specificity of response is generated downstream of a key target, *hairy1*. They show a regional diversity in the properties of neural progenitors and highlight the importance of taking into account the positional origin of stem cells in designing rational strategies to manipulate their proliferative potential.

## Results

### miR-9 Expression Differs along the AP Axis

**Figure 1:**
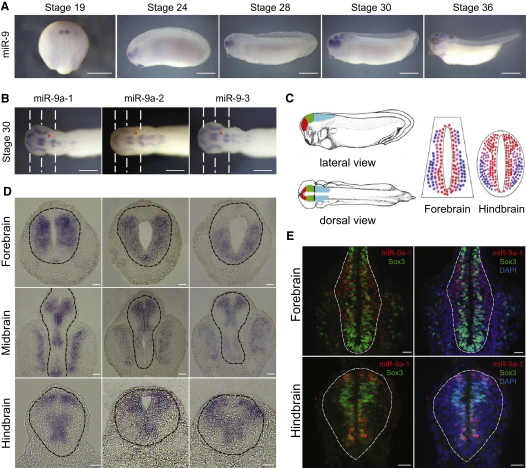
miR-9 Expression Differs along the AP Axis (A) Whole-mount in situ hybridization for miR-9 expression in *X*. *tropicalis* using LNA probe. (B) Expression of miR-9 primary transcripts at stage 30—dorsal view. Dashed line indicates the plane of sectioning in (D). MHB is indicated with an asterisk. Scale bar, 200 μm. (C) Schematic representation of the different regions in the neural tube (red, forebrain; green, midbrain; blue, hindbrain) and transverse sections from the forebrain and hindbrain (red, progenitors; purple, intermediate zone; blue, neurons). (D) In situ hybridization for miR-9 precursors in transverse sections from stage 30 embryos. CNS tissue is outlined with a dashed line. Scale bar, 20 μm. (E) FISH for *miR-9a-1* (in red) combined with immunohistochemistry (IHC) for Sox3 (marker for neural progenitors) in stage 30 embryo. CNS tissue is outlined with a dashed line. DNA is stained with DAPI (4,6-diamidino-2-phenylindole). Scale bar, 20 μm.

First, we examined miR-9 expression during the development of *X*. *tropicalis* using in situ hybridization (miR-9 LNA probe). miR-9 expression was evident in the prospective forebrain region in the anterior neural plate at stage 18/19. At stage 23/24 mature miR-9 was also detected in the developing eye and retina but later on its expression in the neural tube expanded to the more posterior parts of the brain, including the mid- and hindbrain at stage 30–36 (Figure 1A). There are four predicted miR-9 encoding loci in the genome of *X*. *tropicalis*, which give rise to nearly identical mature miR-9 after processing (see Figure S1A available online). Expression of the individual transcripts was similar to the expression of mature miR-9 (Figure 1B; Figure S1B); however, miR-9a-1 was expressed at higher levels than the others. Transcripts were present in the forebrain, the eye, and in the mid- and hindbrain, but no expression was detected in the MHB (Figure 1B; Figure S1B, marked with asterisk), in agreement with reports in the zebrafish (Leucht et al., 2008), and no expression was evident in the spinal cord. We could not detect a signal for miR-9b, consistent with previous results (Walker and Harland, 2008).

During neural development progenitors divide in the VZ, and daughters that exit the cell cycle, migrate laterally to the marginal zone where they differentiate (Figure 1C). Sections showed that miR-9 transcripts have widespread expression in the forebrain but were restricted to the VZ in the more posterior areas (Figure 1D; Figure S1C). These spatial differences became even more apparent later during development (stage 36, Figure S1D). To determine whether miR-9 was also present in post-mitotic neurons in the forebrain or expressed only in progenitors along the AP axis, we used fluorescent in situ hybridization (FISH) for miR-9a-1 combined with immunostaining for Sox3 (marker for neural progenitors) at stages 30 and 36. We found that in the forebrain, miR-9 was transcribed in both Sox3-positive and Sox3-negative cells, whereas it appeared to be restricted to the Sox3-positive domain in the hindbrain (Figure 1E; Figure S1E). This suggests that miR-9 expression differs along the AP axis within a single species and raises the question whether it has the same function in different populations of neural progenitors.

### miR-9 Is Required for Neuronal Differentiation

**Figure 2:**
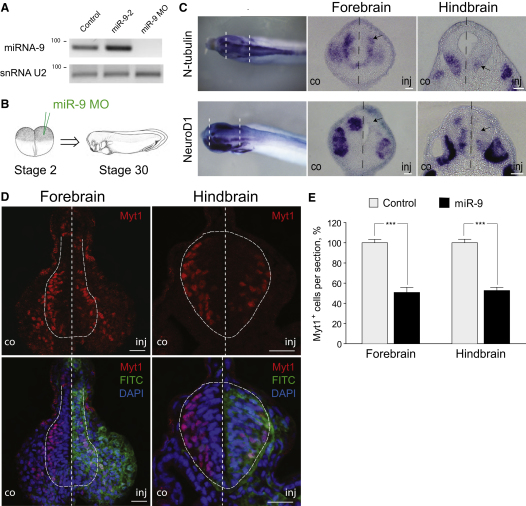
miR-9 Is Required for Neuronal Differentiation (A) Semiquantitative PCR analysis of mature miR-9 levels in stage 30 WT embryos, injected with miR-9-2 precursor or miR-9 MO at one cell stage. The snRNA U2 is used as a loading control. (B) Experimental outline. miR-9 MO was injected in one cell of the two-cell stage embryo, and the injected side was compared to the control at stage 30. (C) In situ hybridization (whole-mount and transverse sections from the forebrain and hindbrain) with markers for differentiated (*N-tubulin*) and differentiating neurons (*NeuroD1*). Note the reduced expression of both markers (arrows) in the miR-9 MO-injected side. (D) Immunohistochemistry on sections for the transcription factor Myt1 indicates impaired neuronal differentiation upon miR-9 knockdown. The FITC tag on miR-9 MO was used to identify the injected side; DAPI was used to stain the DNA. (E) The percentage of Myt1-positive cells in miR-9 MO-injected side relative to the control side in the forebrain (n = 6 embryos, p < 0.001) and hindbrain (n = 9 embryos, p < 0.001). Error bars represent SEM. In all images, scale bars represent 20 μm and CNS tissue is outlined with a dashed line.

In order to gain insight about miR-9's role during neural development, we decided to examine its loss-of-function phenotype. We used an anti-miR-9 specific morpholino (miR-9 MO), which interferes with both the processing of miR-9 precursors and inhibits the activity of the mature miRNA (Kapsimali et al., 2007; Martello et al., 2007) (see Figure S2A for schematic). Injection of miR-9 MO led to an almost complete knockdown of mature miR-9 at early tadpole stage compared to wild-type (WT) embryos, whereas miR-9 levels were increased in embryos injected with miR-9-2 precursor (Figure 2A), as shown using semiquantitative RT-PCR. Knockdown was also confirmed using in situ hybridization and real-time PCR for the mature form of miR-9 (Figures S2B and S2C).

Next, we injected miR-9 MO in one cell of a two-cell stage embryo and compared the injected to the control side at stage 30 at the forebrain and hindbrain level (Figure 2B). Depletion of miR-9 negatively affected neuronal differentiation, as indicated by the decreased expression of *N-tubulin* (n = 14/25) and *NeuroD1* (n = 18/24) (Figure 2C, arrows). The number of Myt1-positive cells (a transcription factor expressed in post-mitotic neurons; Bellefroid et al., 1996) was also reduced in the miR-9 MO-injected side (Figure 2D), but not when control MO was used (Figure S2D). Quantification of the results showed that miR-9 depletion caused a reduction of the number of Myt1-positive cells to about 51% of the control in the forebrain (n = 7 embryos; p < 0.001), and 53% of the control in the hindbrain (n = 9 embryos; p < 0.001) (Figure 2E). These results indicate that miR-9 is required for neuronal differentiation, regardless of the position along the AP axis.

### miR-9 Knockdown Promotes the Proliferation of Neural Progenitors in the Hindbrain

**Figure 3:**
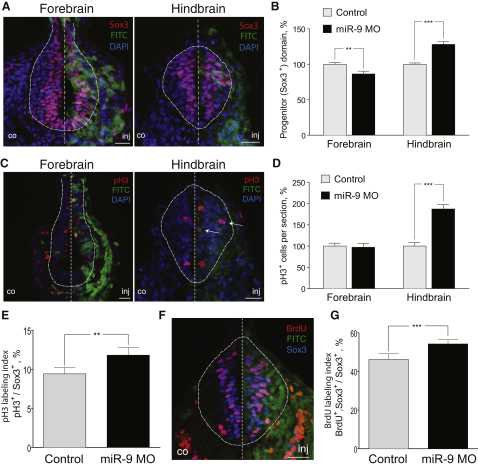
miR-9 Regulates Progenitor Proliferation in a Region-Specific Manner (A) Immunohistochemistry on sections for Sox3 shows expansion of the progenitor domain in the hindbrain. (B) Area occupied by Sox3-positive cells (progenitor domain) per section in miR-9 MO-injected side expressed relative to the control side in the forebrain (n = 7, p = 0.008) and hindbrain (n = 9, p < 0.001). (C and D) Transverse sections from the forebrain or hindbrain of miR-9 MO-injected embryos analyzed for the mitotic marker pH3 show a hindbrain-specific increase in the number of mitotic cells (n = 11, p < 0.001), but no change in the forebrain (n = 9). (E) pH3-labeling index (pH3^+^ cells over Sox3^+^ cells) in the hindbrain (n = 6, p = 0.004). (F and G) Rate of proliferation of the hindbrain progenitors is increased, as determined by BrdU incorporation for 30 min. BrdU-labeling index is calculated as the percentage of BrdU^+^ and Sox3^+^ cells over the total population of Sox3^+^ cells (n = 7, p < 0.001). In all panels, scale bars represent 20 μm, FITC staining shows the MO-injected side, DNA was counterstained with DAPI, CNS tissue is outlined with a dashed line, and error bars represent SEM.

We hypothesized that miR-9 depletion could interfere with the onset of the neurogenic program by preventing cell-cycle exit, resulting in an increase in the number of progenitors. To test this we measured the area occupied by Sox3-positive neuronal progenitors per section in miR-9 MO-injected embryos. As expected, in the hindbrain there was an increase of the progenitor domain by 28% compared to the control (n = 9; p < 0.001) (Figures 3A and 3B). However, in the forebrain the Sox3-positive area was not increased, and if anything it was slightly decreased by 14% compared to the control (n = 7; p = 0.008). In the hindbrain some Sox3-positive cells were found further away from the ventricle (data not shown), thus found in positions where differentiated cells would normally reside.

**Figure 4:**
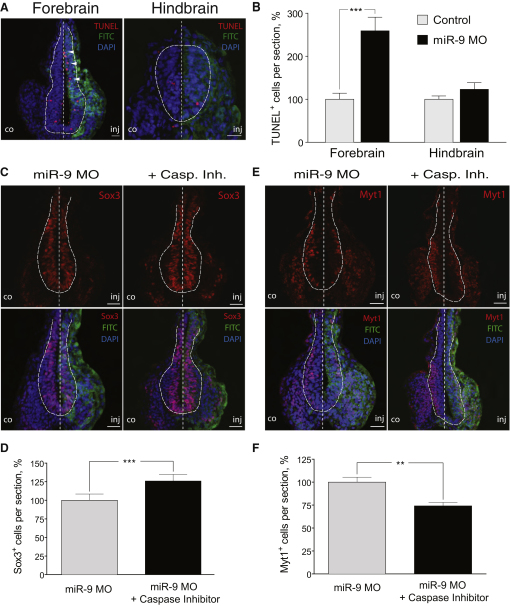
miR-9 Depletion Negatively Affects the Survival of Forebrain Neural Progenitors (A) TUNEL staining shows increased apoptosis upon miR-9 depletion in the forebrain (arrowheads), but not in the hindbrain. (B) Percentage of the TUNEL^+^ cells in the injected compared to the control side in the forebrain (n = 6, p < 0.001) and in the hindbrain (n = 6). Error bars represent SEM. (C and D) Sox3-positive domain is expanded in miR-9 MO-injected side when apoptosis is prevented (n = 7, p < 0.001). (E and F) The reduced number of differentiating neurons (Myt1^+^) upon miR-9 depletion is not rescued by caspase inhibitor block of apoptosis (n = 6, p = 0.003). In all images FITC staining shows the MO-injected side; DNA was counterstained with DAPI. Neural tube is outlined with a dashed line. Scale bars, 20 μm. Error bars represent SEM.

To find out if there was a corresponding increase in the number of cells undergoing mitosis, we examined the number of phospho-histone H3 (pH3)-positive cells in both areas. miR-9 knockdown led to an almost 2-fold increase in the number of pH3-positive cells in the hindbrain, but there was no apparent change in the forebrain (Figures 3C and 4D, p < 0.001). Injection of control MO had no effect on either Sox3 or pH3 expression (Figures S2E and S2F). To examine whether the increase in the Sox3-positive and pH3-positive cells was due to a change in cell proliferation, we performed double labeling for pH3 and Sox3 and found that the labeling index (pH3^+^/Sox3^+^ cells in the hindbrain) is increased upon miR-9 knockdown (Figure 3E, p < 0.01). The increased rate of proliferation of the hindbrain progenitors was also confirmed using BrdU labeling of the proliferating progenitors (Figures 3F and 3G, p < 0.001).

These observations suggest that miR-9 function in the hindbrain is important for limiting progenitor proliferation and promoting the onset of the neurogenic program and raises interesting questions about how (and why) that differs in the forebrain.

### miR-9 Depletion Causes Apoptosis in Forebrain Progenitors

One possibility for the decrease of differentiated neurons in the forebrain is increased apoptosis. Indeed, TUNEL analysis showed that miR-9 MO caused an increase in apoptosis in the forebrain, which was specific for that area, and it was not observed in the hindbrain (Figures 4A and 4B, p < 0.001). Apoptotic cells were present throughout the forebrain but were most frequent in the VZ (Figure 4A, arrows). No increase in apoptotic cells was apparent when control MO was used (Figure S2G).

An important question is whether the cells undergoing apoptosis represent neuronal progenitors or differentiating neurons. Because miR-9 knockdown caused only a modest reduction of the progenitor domain but a significant decrease in the number of neurons (see Figure 2), one may hypothesize that it is the forebrain neurons that undergo apoptosis in the absence on miR-9. Alternatively, miR-9 depletion could reduce the survival of the forebrain progenitors, which would be consistent with the location of the majority of the apoptosing cells (see above). In order to distinguish between these possibilities, we blocked cell death by injecting a pan-caspase inhibitor together with miR-9 MO or control MO. Cell death was efficiently prevented, as evident by the reduction of the number of apoptotic cells compared to injecting miR-9 MO alone (Figure S2H). Coinjection of caspase inhibitor together with miR-9 MO led to an expansion of the Sox3-positive area in the forebrain, compared to miR-9 MO alone (Figures 4C and 4D), whereas the number of differentiating neurons was still reduced (Figures 4E and 4F). Effectively, preventing apoptosis made the miR-9 loss-of-function phenotype in the forebrain more similar to the one observed in the hindbrain. Taken together, this suggests that miR-9 is necessary for the transition of progenitors to neurons across the AP axis, and in addition it is required for the survival of progenitors in the forebrain.

### *hairy1* Is an Endogenous Target of miR-9 In Vivo

To understand how the differences in miR-9 loss-of-function phenotype along the AP axis arise at molecular level, we set to determine the potential miR-9 targets in *X*. *tropicalis* in relation to the phenotype we observed. One possibility was that miR-9 might regulate two or more regionally restricted targets, which in turn mediate functional specificity in different areas of the CNS. Alternatively, miR-9 specificity of function might be generated downstream of one primary target, which is expressed along the AP axis but has different functions in different axial levels (Figure S3A).

As a starting point we used bioinformatic analysis using the overlap of the targets predicted by the algorithms PicTar (Krek et al., 2005) and TargetScan (Lewis et al., 2003) to identify more than 500 potential miR-9 targets based on target site conservation in mammals (data not shown). This data set was further refined using GO analysis (Figure S3B) conservation of the seed in *Xenopus* (data not shown), luciferase reporter assay in HeLa cells (Figures S3D and S3E), and whole-mount in situ hybridization expression screen (Figure S3F). We decided to focus on the members of the hes (hairy and enhancer of split) family, which have been shown to play crucial roles in maintaining neural progenitors (Baek et al., 2006; Ohtsuka et al., 2001) Among them, *Hes1* was present in all three GO categories, its *Xenopus* homolog *hairy1* showed a prominent effect in the reporter assays, and was also expressed in the CNS, which is why we decided to examine it further.

**Figure 5:**
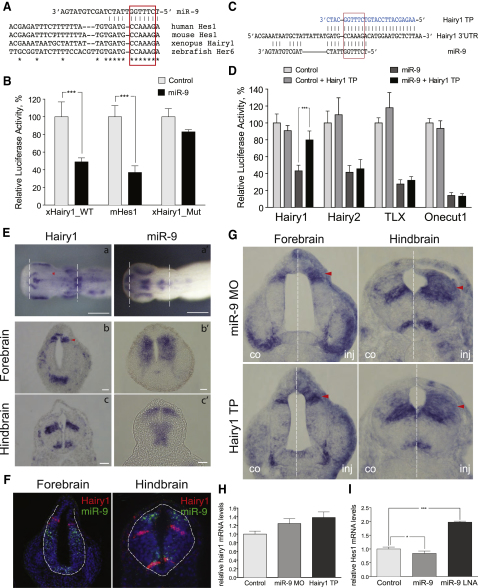
miR-9 Regulates the Expression of *hairy1* In Vivo (A) Sequence alignment of the predicted miR-9 binding site in *HES1* homologs in human, mouse, *Xenopus*, and zebrafish. Positions that have a single, fully conserved residue are marked with an asterisk. Seed-complementary region is boxed in red. (B) HeLa cells were transfected with WT *Xenopus* hairy1 (xhairy1_WT), mouse Hes1 (mHes1), or mutant hairy1 (xHairy1_Mut) reporter together with either scrambled (Control) or miR-9 precursors (miR-9). Luciferase expression was normalized and expressed relative to the control levels. Error bars represent SD. (C) Design of target protector morpholino (Hairy1 TP) directed against *hairy1* miR-9 binding site. Seed region is boxed in red. (D) Hairy1 TP alleviates the repression of hairy1 luciferase reporter when cotransfected with miR-9 precursors but has no effect on the repression of other miR-9 targets. Error bars represent SD. (E) In situ hybridization for miR-9 (miR-9a-1 transcript) and *hairy1* in stage 30 embryos. Shown are whole mounts and transverse sections through the respective brain areas. (F) Double-fluorescent in situ for hairy1 (red) and miR-9a-1 (green) shows mutually exclusive pattern of expression along the AP axis. (G) miR-9 MO and hairy1 TP lead to expansion of the hairy1-positive domain (red arrowheads) along the AP axis, as shown by in situ hybridization. (H) Quantification of the change in hairy1 mRNA expression using qRT-PCR. (I) Hes1 mRNA levels in N1E neuroblastoma cells are downregulated when miR-9 is overexpressed and increased when it is knocked down using LNA inhibitors. In all graphs, data are presented as mean values, and error bars represent SEM.

The *X*. *tropicalis hairy1* is most closely related to the mammalian *Hes1* based on sequence conservation (72%) (Jouve et al., 2000; data not shown). miR-9 binding site is highly conserved in the vertebrate homologs of Hes1, with 100% sequence homology in the seed-complementary region (Figure 5A). In order to test whether miR-9 regulates hairy1 in vitro, we tested *Xenopus* hairy1 (xHairy1) and mouse Hes1 (mHes1) using luciferase-based reporter assay. Both xhairy1 3′UTR (xHairy1-WT) and mHes1 3′UTR were significantly repressed by synthetic miR-9 precursors, whereas this effect was absent when a mutant reporter lacking the seed-complementary sequence (xHairy1_Mut) was used. In order to validate the specificity of the repression, we used a target-protector approach to block miR-9 binding site (Choi et al., 2007). A hairy1 target protector morpholino (hairy1 TP) was designed to overlap with the seed-complementary sequence on hairy1 and extend further in the 3′ direction to confer specificity (Figure 5C). Next, we examined the efficiency and specificity of hairy1 TP. Luciferase reporter assays confirmed that hairy1 TP is able to partially alleviate the repression of miR-9 on the hairy1 luciferase reporter when introduced in vitro together with miR-9 mimics, but not of a reporter carrying the 3′UTR of other miR-9 targets such as *hairy2*, *TLX*, and *Onecut1* (Plaisance et al., 2006) (Figure 5D). These results show that miR-9 is able to repress hairy1 in vitro.

### *hairy1* and miR-9 Expression Is Mutually Exclusive

To gain insight into the miR-9-hairy1 interaction, we compared their expression in vivo. Hairy1 has been cloned from *Xenopus* before (Palmeirim et al., 1997), but here we described its expression in the nervous system in detail. During early brain development (stages 21–26), *hairy1* is expressed in a broad region in the forebrain (data not shown) but later becomes restricted to the roof plate and an intermediate patch of progenitors, which represents the zona limitans intrathalamica (ZLI)—a boundary region between the thalamus and the prethalamus (Figure 5Eb). In this region *hairy1* expression overlaps with the known marker of the ZLI *Shh* (Ishibashi and McMahon, 2002) and is immediately adjacent to the expression of *Irx3*, which marks the thalamic region in chick and mouse (Kiecker and Lumsden, 2004) (Figure S4A). Conversely, in the more posterior areas, hairy1 transcripts are present ventrally in the midbrain but are absent from the mid- and hindbrain boundary, contrary to the expression of *Hes1* in the mouse and the hairy-related genes *her5/9* in zebrafish. In the hindbrain *hairy1* expression is restricted to distinct domains—in a ventral region adjacent to the floor plate and in an intermediate region of progenitors (Figure 5Ec). Mammalian *Hes1* is also expressed at high levels in the ZLI and in an intermediate zone of progenitors in the hindbrain (Baek et al., 2006), but in addition it is also expressed throughout the VZ in the telencephalon and in the boundary regions such as MHB, the roof plate, and the floor plate. The zebrafish *her5* is also expressed in boundary regions such as the MHB (Geling et al., 2003). Thus, *Xenopus tropicalis hairy1* shows similarities and differences with *hes1*; both are expressed in the ZLI but unlike *hes1*, *hairy1* is not expressed in the roof plate or the floor plate or the MHB, with the exception of the roof plate in the forebrain. Instead, *Hairy1* is expressed in a subset of dorsoventrally restricted progenitors within the neurogenic compartments. This expression pattern appears complementary to that of mir-9 in whole mounts and sections (Figure 5E). Double FISH for miR-9a-1 and hairy1 confirmed that their expression is mutually exclusive along the AP axis with the exception of a few double-stained cells in the ventral hindbrain (Figure 5F).

### miR-9 Regulates hairy1 In Vivo

In order to determine whether miR-9 regulates hairy1 in vivo, we examined hairy1 expression in morphant embryos using in situ hybridization. Both miR-9 MO (n = 22/36) and hairy1 TP (n = 20/35) led to an expansion of the hairy1-positive domain along the AP axis: in the forebrain the expression in the roof plate and in the ZLI region was expanded, whereas posteriorly the hairy1-positive domain expanded both laterally and dorsally (Figure 5G).

The expansion of the expression domain of Hairy1 suggests that miR-9 acts at the mRNA level. Indeed, miR-9 MO and hairy1 TP led to an increase in hairy1 mRNA levels, as shown by real-time PCR (Figure 5H). In addition, miR-9 overexpression in a neuroblastoma cell line (N1E-115) decreased the RNA level of the murine homolog, *Hes1*, and conversely, inhibition of endogenous miR-9 with miR-9 LNA increased it (Figure 5I). These findings suggest that that the mechanism of miR-9 regulation is evolutionarily conserved and that miR-9 acts by destabilizing the mRNA rather than repressing protein translation. This is consistent with recent reports that contrary to what was previously thought, decreasing mRNA levels is the main mode of repression for mammalian microRNAs (Guo et al., 2010).

### Hairy1 TP Functionally Mimics miR-9 MO Phenotype

**Figure 6:**
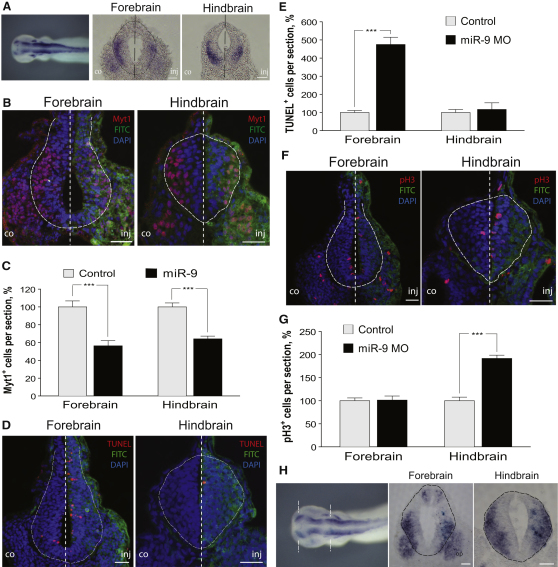
Hairy1 Target Protector Mimics miR-9 MO Phenotype (A) In situ hybridization (whole-mount and transverse sections from the forebrain and hindbrain) for *N-tubulin* in hairy1 TP-injected embryos. (B) The number of differentiating neurons (Myt1^+^ cells) is decreased upon injection of hairy1 TP. (C) Quantification of the Myt1^+^ cells in the forebrain (n = 7, p < 0.001) and the hindbrain (n = 7, p < 0.001). Myt1^+^ cells in the injected side were expressed as a percentage of the control side. (D) Hairy1 TP leads to forebrain-specific induction of apoptosis as indicated by TUNEL staining. (E) Quantification of the TUNEL-positive nuclei in the forebrain (n = 7, p < 0.001) and in the hindbrain (n = 5). (F) Immunostaining for pH3 in embryos injected in one side with hairy1 TP. (G) Relative number of pH3^+^ cells in the hairy1 TP-injected compared to the control side in the forebrain (n = 11) and the hindbrain (n = 9, p < 0.001). (H) In situ hybridization for *N-tubulin* (purple) in embryos electroporated in one side with hairy1 Δ3′UTR and lacZ DNA as a tracer. Light-blue staining indicates the electroporated area. op, olfactory placodes. Scale bars, 20 μm. In all panels, FITC was used to identify the MO-injected side; DNA was counterstained with DAPI; CNS tissue is outlined with a dashed line; and error bars represent SEM.

In order to determine the contribution of *hairy1* repression to miR-9 function, we examined the effect of hairy1 TP on neuronal differentiation, progenitor proliferation, and apoptosis. Injection of hairy1 TP resulted in decrease in the expression of *N-tubulin* (Figure 6A, n = 11/17), whereas TP designed against another potential miR-9 target—*NR2E1/TLX* had no effect on *N-tubulin* expression (data not shown). Furthermore, the number of Myt1-positive cells was also negatively affected in both the forebrain and the hindbrain (Figures 6B and 6C). As with the miR-9 MO, neuronal reduction was accompanied by an increase in apoptotic cells in the forebrain (Figures 6D and 6E) and an increase in proliferating cells in the hindbrain (Figures 6F and 6G). Furthermore, electroporation of hairy1 construct lacking the 3′UTR together with LacZ DNA as a tracer led to a reduction in *N-tubulin* expression (Figure 6H, n = 16/18 embryos), confirming the ability of hairy1 to repress the neurogenic program in both areas. Electroporation of LacZ alone had no effect on *N-tubulin* expression (data not shown).

These results show that alleviation of miR-9 repression on hairy1 mimics miR-9 MO phenotype and suggest that posttranscriptional regulation of *hairy1* is one of the essential aspects of miR-9 function during neural development. They also point out that the specificity of miR-9 function is generated downstream of *hairy1*.

### Changes in *Cyclin D1* and *p27Xic1* Expression Contribute to Increased Progenitor Proliferation

**Figure 7:**
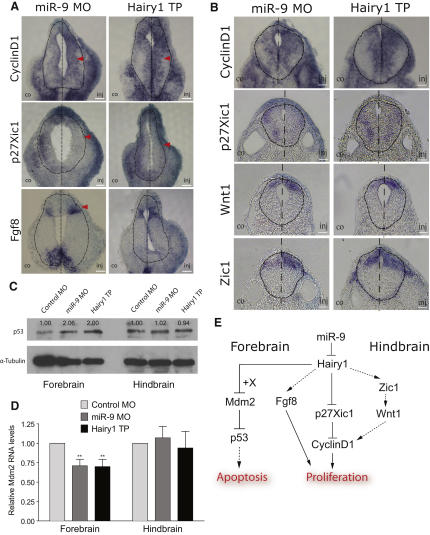
Mechanism of miR-9 Function (A) Forebrain sections of miR-9 MO or hairy1 TP-injected embryos analyzed for *CyclinD1*, *p27Xic1*, and *Fgf8* expression by in situ hybridization. (B) Hindbrain sections of miR-9 MO or hairy1 TP-injected embryos analyzed for *CyclinD1*, *p27Xic1*, *Wnt1*, and *Zic1* expression. (C) Representative western blot for endogenous p53 protein levels in forebrain or hindbrain tissue isolated from *X*. *tropicalis* embryos injected with control MO, miR-9 MO, or hairy1 TP. Numbers represent the mean from three experiments, 60 embryos each. (D) Real-time PCR analysis for *Mdm2* expression, normalized for the ribosomal protein RPL8 (n = 3 experiments, 20 embryos each). Error bars represent SEM. (E) Model for miR-9 function in cell survival and progenitor proliferation.

To understand the mechanism by which miR-9 affects proliferation and apoptosis, we looked at molecular pathways that may be regulated by miR-9 through *hairy1*. Injection of either miR-9 MO or hairy1 TP led to an expansion of the expression domain of *cyclin D1* (miR-9 MO: n = 18/28 embryos; n = 15/24 hairy1 TP), which promotes G1-S phase progression and to the downregulation of *p27Xic1* expression, a cyclin-dependent kinase inhibitor (miR-9 MO, 12/19; hairy1 TP, 10/19). This was observed both in the hindbrain and the forebrain (Figures 7A and 7B, arrowheads), consistent with an effect on proliferation on both areas. In mammals, *p27* has been shown to be a direct target of Hes1 (Murata et al., 2005); therefore, the interaction of hairy1 with *p27Xic1* is likely to be direct. By contrast the upregulation of *cyclin D1* by *hairy1* is likely to be indirect (diagram in Figure 7E). *Cyclin D1* is a direct downstream target of *Wnt1* (Megason and McMahon, 2002), which was also increased in the injected side (miR-9 MO, eight of 14; hairy1 TP, ten of 18) (Figure 7B). *Hairy1* may affect *Wnt1* expression through *Zic1*, a transcription factor known to promote the proliferation of neural progenitors (Aruga et al., 2002; Elsen et al., 2008). *Zic1* positively regulates wnt signaling both in *Xenopus* and zebrafish (Elsen et al., 2008; Merzdorf and Sive, 2006), and in addition another member of the hes family in *Xenopus*, *hairy2*, has been previously shown to regulate *Zic1* (Nichane et al., 2008). Therefore, it is very likely that *Zic1* lies between *hairy1* and *wnt1*. Indeed, injection of either miR-9 MO (n = 12/20) or hairy1 TP (n = 10/23) led to a lateral expansion of the *Zic1* domain in the hindbrain (Figure 7B), suggesting that *Zic1* may mediate the *hairy1* regulation on wnt1 pathway. However, we cannot rule out the possibility that miR-9 might affect wnt signaling independently of *Zic1* or that it can be involved in the regulation of other signaling pathways (such as BMP signaling) in addition to wnt.

In the forebrain, *wnt1* is not expressed, and *Zic1* expression is not affected by miR-9 knockdown (data not shown); therefore, the effect of *hairy1* on proliferation may be mediated via an intermediate regulator other than *wnt*. Fgf signaling is known to promote proliferation in the developing forebrain (Storm et al., 2006), and *Fgf8* is expressed in the ZLI area (Kataoka and Shimogori, 2008). Using double FISH, we found that *hairy1* overlaps with *Fgf8* (Figure S4C) and furthermore, injection of miR-9 MO (n = 8/14) and hairy1 TP (n = 10/18) led to an expansion of the *Fgf8*-positive domain (Figure 7A). Therefore, even though *hairy1* is expressed in a restricted domain, it regulates neurogenesis throughout the neural tube via non-cell-autonomous signaling pathways.

### p53 Contributes to miR-9 MO-Induced Apoptosis in the Forebrain

To understand the molecular pathway behind the differential effects on miR-9 on apoptosis in the forebrain versus the hindbrain, first, we examined p53 expression in these two areas because p53 has been shown to mediate Notch-induced apoptosis in the forebrain (Yang et al., 2004). Injection of either miR-9 MO or hairy1 TP led to approximately 2-fold increase in p53 protein levels in the forebrain, but not in the hindbrain (Figure 7C, n = 3 experiments, 60 embryos each). This correlated well with the region-specific induction of apoptosis we observed in the embryo using TUNEL (Figures 4A and 4B) and suggests that activation of p53 pathway may be responsible for this phenotype.

We examined whether the upregulation of p53 is mediated through its regulator *Mdm2* (Haupt et al., 1997) using quantitative RT-PCR. We found that miR-9 MO or hairy1 TP led to an approximately 30% decrease in *Mdm2* mRNA expression in the forebrain but did not significantly affect *Mdm2* levels in the hindbrain (Figure 7D, n = 3 experiments, 20 embryos each). Hes1 has been previously shown to activate the p53 pathway through *Mdm2*; however, this probably requires specific cofactors because Hes1 cannot bind *Mdm2* promoter per se (Huang et al., 2004). Nevertheless, the lack of *mdm2* repression by *hairy1* in the hindbrain provides a molecular explanation for the lack of an effect on apoptosis in this region when miR-9 is depleted.

## Discussion

In this study we have examined the role of miR-9 during *Xenopus* neurogenesis, focusing on the regional differences in its expression and function. Conflicting results about miR-9 expression and function in different model systems have been obtained, even though its sequence is 100% conserved (Delaloy et al., 2010; Leucht et al., 2008; Zhao et al., 2009). In regard to expression, previous studies were either based mainly on the location of the expressing cells in relation to the VZ or did not examine different AP levels (Delaloy et al., 2010; Deo et al., 2006; Leucht et al., 2008; Shibata et al., 2008). Here, by comparison to other markers, we have shown that miR-9 expression differs along the AP axis, even in a single species—it is expressed in both neurons and progenitors in the forebrain but becomes restricted to progenitors in the more posterior brain regions, namely the midbrain and hindbrain.

In addition to regional differences in expression, our work has uncovered a regional difference in the function of miR-9 in progenitor cells. Using a loss-of-function approach, we have found that in the absence of miR-9, neurogenesis fails along the AP axis. At the same time, in miR-9 MO embryos the number of progenitors increases in the hindbrain, but paradoxically, it slightly decreases in the forebrain. However, an underlying increase in forebrain progenitors is uncovered when apoptosis is blocked. We propose that miR-9 is necessary for cell-cycle exit throughout its AP domain of expression, but neuronal progenitors in the forebrain additionally and uniquely require miR-9 for their survival. Therefore, in the forebrain, in the absence of miR-9, extra proliferation is counterbalanced by increased apoptosis, resulting in no net increase in the number of forebrain progenitors, and even a slight decrease. Such context-dependent activity of miR-9 based on the regional identity of progenitor cells may explain previously conflicting results with respect to miR-9 function (Delaloy et al., 2010; Zhao et al., 2009).

To our knowledge, an effect on apoptosis in neural development by miR-9 knockdown has not been reported before. However, Dicer ablation in the mouse forebrain led to increased cell death in committed neuronal progenitors (De Pietri Tonelli et al., 2008), and miR-24a is required to prevent apoptosis in the retina (Walker and Harland, 2009). Our studies have not revealed a function of miR-9 in forebrain neurons because their formation is mostly prevented by the loss of function. Alternative strategies will be needed to address this question.

Having shown distinct effects on proliferation and apoptosis, an important question is whether these are mediated by one primary miR-9 target or the coordinate regulation of several targets. Theoretically, microRNAs are capable of regulating many target genes, and miR-9 is no exception to this. Indeed, our bioinformatic analysis followed by luciferase assay verification identified several genes as potential miR-9 targets. Similarly, previous reports in other species have identified several miR-9 targets. In the fish, several components of the FGF pathway and *Her5* have been proposed as targets involved in the formation of the MHB and *Her9* in the control of neurogenesis (Leucht et al., 2008). In the mouse, proposed targets include *FoxG1* in the developing mouse telencephalon (Shibata et al., 2008), *NR2E1/TLX* in adult neural stem cells (Zhao et al., 2009), and *stathmin* in human embryonic stem cell-derived neural progenitors (Delaloy et al., 2010). However, with the exception of *Her5*, target-protector experiments (Leucht et al., 2008), where the endogenous putative target is specifically protected from miR-9 binding, have not been performed; therefore, it is very difficult to evaluate the contribution of these targets to the miR-9 loss-of-function phenotype.

In our work, Hairy1 target-protector experiments recapitulated the miR-9 MO phenotype in vivo, including the regional-specific effects in apoptosis. These results suggest that a single target, *hairy1*, mediates the effects of miR-9 on neurogenesis, proliferation, and apoptosis. In this scenario the regional specificity of function is regulated downstream of Hairy1, rather than directly downstream of miR-9. Although we cannot exclude the possibility that other targets mediate other aspects of miR-9 activity, our results suggest that miR-9 falls into the growing category of miRNAs that have just one or few important targets, although many more can be bioinformatically predicted (reviewed in Flynt and Lai, 2008). Such miRNAs tend to be involved in “developmental genetic switching” rather than “fine tuning,” a hypothesis that is consistent with the proposed role of miR-9 in neurogenesis.

What is the significance of *hairy1* as a miR-9 target? Hairy1 is a member of the hes (hairy and enhancer of split), helix-loop-helix (bHLH) type transcriptional repressors. Several Hes genes, such as *Hes1, Hes3* in the mouse and the *her5* in zebrafish, are expressed at high levels in boundary of the nervous system (Baek et al., 2006). Such boundary regions, exemplified by the ZLI, the MHB, the floor, and roof plate, are characterized by secretion of morphogens, slow proliferation of progenitors, and lack of neurogenesis. *Hes1* is also expressed at variable levels in adjacent neural compartments where neurogenesis actively takes place. These expression data and the results of functional analysis gave rise to a model whereby the high persistent levels of Hes1 observed in boundaries suppress neurogenesis, whereas in compartments the variable levels permit neurogenesis when protein levels are low (Baek et al., 2006). The variable *Hes1* levels are in fact oscillatory (Shimojo et al., 2008), and such oscillations are thought to be driven both by mRNA and Hes1 protein instability, although factors that mediate the mRNA instability are not known (Davis et al., 2001; Hirata et al., 2002; Shimojo et al., 2008). It is tempting to speculate that miR-9 is involved in hairy1/Hes1 oscillations by regulating mRNA stability because, indeed, the role of mRNA stability in the Hes1 oscillator has been previously theoretically predicted (Hirata et al., 2002; Xie et al., 2007). This would be consistent with the expression of both miR-9 and *Hes1* in proliferating progenitors in the VZ of the mammalian telencephalon (Baek et al., 2006; Delaloy et al., 2010) and our observations that there is conserved miR-9 binding site in the 3′UTR of Hes1, and that both hairy1 and hes1 are regulated by miR-9 at mRNA level. Because Hairy1 is a primary target of miR-9, regional specificity is generated downstream of *hairy1*, culminating in the differential effect on apoptosis in the forebrain versus the hindbrain. In turn, this specificity may be mediated by the presence, availability, or activity of cofactors, some of which may be tissue specific. Indeed, several cofactors for the Hes family of genes have been identified, such as Id and Groucho (Bai et al., 2007; McLarren et al., 2001).

To summarize, we propose that in normal development, miR-9 promotes neurogenesis by lowering the levels of *hairy1* such that cells can exit the proliferative compartment. In the absence of miR-9, *hairy1* levels remain high, and progenitor cells cannot complete the differentiation program. A regional specificity of action is evident in that forebrain progenitors that fail to exit the cell cycle undergo apoptosis. Therefore, in the forebrain the proliferative effect of miR-9 depletion can only be seen when apoptosis is also blocked. These findings complement the miR-9/ her5 regulation in the zebrafish MHB (Leucht et al., 2008) and show that miR-9 regulation of hairy genes is more widespread, occurring well outside boundary regions.

Our results have far-reaching implications for any cancer therapies and stem cell expansion that rely on manipulating miR-9 levels. In terms of stem cell expansion, their positional identity may determine whether they will undergo proliferation or apoptosis in response to blocking miR-9. On the other hand, an inhibitor of miR-9 may have therapeutic potential in forebrain-derived tumors, inducing apoptosis of progenitors, but may have an undesirable effect in tumors of hindbrain origin, enhancing their proliferation. The regional-specific effect of miR-9 on neural progenitors underscores the importance of taking into account the positional identity of cells when testing miR-9 function in normal development and disease.

## Experimental Procedures

### DNA Constructs and Electroporation

For the generation of luciferase reporter constructs, 3′UTR of predicted miR-9 targets (or 1 kb region containing the seed-complementary sequence if the 3′UTR was not annotated) was PCR amplified from *X*. *tropicalis* genomic DNA and cloned downstream of Renilla luciferase coding sequence in the psiCHECK-2 vector (Promega). miR-9-2 WT and miR-9-2 Mut were amplified from genomic DNA as described previously (Shibata et al., 2008) and cloned in the pCS2+ vector. pCS2-Hairy1 construct lacking the 3′UTR was electroporated together with LacZ DNA as a tracer into the brain of stage 26 embryos using SD9 stimulator (Grass Technologies) as previously described (Falk et al., 2007).

### Morpholino Design and Injection

The anti-miR-9 morpholino (5′-CTCATACAGCTAGATAACCAAAGAT-3′), the hairy1 target protector morpholino (5′-AAGAGCATTCCATGTCTTTGGCATC-3′), and the standard Negative Control Morpholino (5′-CCTCTTACCTCAGTTACAATTTATA-3′) were purchased from Gene Tools LLC and used at the following amounts: control MO (one side, 10 ng; whole embryo, 20 ng); miR-9 MO (one side, 7.5 ng; whole embryo, 15 ng); and hairy1 TP (one side, 10 ng; whole embryo, 20 ng). All morpholinos were conjugated to FITC, and the injected side was identified using primary mouse anti-FITC (1:250; Roche) and anti-mouse Alexa 488 (1:500; Molecular Probes) antibodies.

### In Situ Hybridization

Whole-mount in situ hybridizations were performed as previously described (Bourguignon et al., 1998). Mature miR-9 was detected using miR-9 DIG-labeled LNA probe (TCATACAGCTAGATAACCAAAGA; Exiqon) and the following modifications to the standard in situ protocol: additional fixation using 1-ethyl-3-(3-dimethyl-aminopropyl) carbodiimide for 1 hr (adapted from Pena et al., 2009) and hybridization temperature 52°C. Fluorescent in situ hybridization (FISH) was performed as previously described (Vize et al., 2009) with the following modification - signal was detected using tyramide signal amplification (Perkin Elmer). Detailed protocols are available upon request. Neural tube boundary was drawn based on high-magnification DAPI or bright-field images.

### Cryosectioning, Antibody Staining, and Immunoblotting

For immunohistochemistry, embryos fixed in MEMFA (0.1 M MOPS [pH 7.4], 2 mM EGTA, 1 mM MgSO_4_, 3.7% formaldehyde) were sectioned on a Leica CM3050 S cryostat after embedding in 25% fish gelatin/15% sucrose and stained as described previously (Chalmers et al., 2003; Regad et al., 2007). The following primary antibodies were used: anti-Sox3 (1:2000; gift from Klymkovsky laboratory); anti-Myt1 (1:1000; Sabherwal et al., 2009); anti-pH3 (1:500; Upstate); and anti-p53 (1:1000; Abcam). Appropriate secondary antibodies were obtained from Molecular Probes.

For western blot, primary mouse anti-p53 (1:100; Abcam), mouse anti-α-tubulin (1:5000; Sigma), and secondary anti-mouse HRP (1:2000; DakoCytomation) were used. Experiment was repeated three times (with 60 embryos each), and results were quantified using Intelligent Quantifier software (Bio Image Systems).

### TUNEL Staining and Apoptosis Inhibitor

TUNEL staining was performed using TMR red In Situ Cell Death Detection kit according to the manufacturer's instructions (Roche). TUNEL-positive cells were counted across two consecutive sections in at least six embryos and averaged. Values were expressed relative to the number of apoptotic cells in the control side.

Apoptosis was blocked using a pan-caspase inhibitor (Z-VAD (OMe)-FMK; Calbiochem), which was injected at two-cell stage at a final concentration of 2 ng/μl.

### RNA Isolation, RT-PCR, and Quantitative Real-Time PCR Analysis

Total RNA was extracted from either whole embryos or forebrain/hindbrain tissue using TRIzol (Invitrogen) and retrotranscribed using RT-AMV (Invitrogen) according to the manufacturer's instructions. Mature miR-9a levels were assessed using modified semiquantitative RT-PCR as previously described (Martello et al., 2007). Quantitative real-time PCR was performed in an ABI StepOnePlus Sequence Detection System (Applied Biosystems) using TaqMan Fast Real-Time PCR Master Mix and probes purchased from Applied Biosystems. The expression of *X*. *tropicalis* genes was normalized for *Rpl8*, whereas Hes1 expression was normalized to *Gapdh* in mouse. miR-9 expression was examined using TaqMan microRNA assay (ABI).

### Cell Culture and Luciferase Reporter Assay

HeLa cells were maintained in DMEM supplemented with 10% serum and antibiotics. N1E-115 neuroblastoma cell line was obtained from ECACC and maintained in DMEM supplemented with 10% serum and GlutaMAX (Invitrogen). For *Hes1* expression analysis 24 hr after transfection with miR-9 precursors (30 nM) or miR-9 LNA inhibitor (50 nM), cells were synchronized by serum starvation as previously described (Hirata et al., 2002). For luciferase reporter assays, cells were seeded at a density of 10^4^ cells/well in a 96-well plate and transfected after 24 hr with 25 ng of the reporter and either 30 nM of scrambled or miR-9 precursors (Ambion). Luciferase expression was analyzed after 48 hr using Dual Luciferase Assay system (Promega). Renilla luciferase activity was normalized by the coexpressed Firefly Luciferase and expressed as a percentage of the control. All assays were repeated at least three times and performed in triplicate each time.

### Statistical Analysis

For Myt1, pH3, or TUNEL analysis, positive cells were counted across two consecutive sections in the corresponding brain area and the numbers averaged per embryo. Sox3 expression was quantified by drawing a border around the area containing Sox3-positive cells and measuring the area using ImageJ. Values were expressed relative to the control side. N numbers represent number of embryos from at least three experiments unless otherwise indicated. Statistical analysis of the data (two-tailed unpaired Student's t test, calculation of SEM) was done using SigmaStat 3.0 (Aspire Software). Statistical significance is indicated as follows: ^∗^p < 0.1, ^∗∗^ p < 0.01, ^∗∗∗^ p < 0.001.

## Supplemental Information

Document S1. Four Figures
