# Supplementary material for: MicroRNA-9 Reveals Regional Diversity of Neural Progenitors along the Anterior-Posterior Axis
Source: Dev Cell. 2011 Jan 18;20(1):19–32. doi: 10.1016/j.devcel.2010.11.018 (PMC3361082; doi:10.1016/j.devcel.2010.11.018)
Supplement: Document S1. Four Figures [file mmc1.pdf]

Developmental Cell, Volume *20*

Supplemental Information

MicroRNA-9 Reveals Regional Diversity of Neural Progenitors along the Anterior-Posterior Axis

Boyan Bonev, Angela Pisco, and Nancy Papalopulu

Figure S1. miR-9 expression in *Xenopus Tropicalis* - relates to Figure1

Figure S2. Injection of control morpholino has no effect on neural development – relates to Figure 2, 3 and 4

Figure S3. Target screen identified several miR-9 targets – relates to Figure 5

Figure S4. *Hairy1* is expressed in the ZLI boundary region and overlaps with *Fgf8* domain – relates to Figure 5 and 7

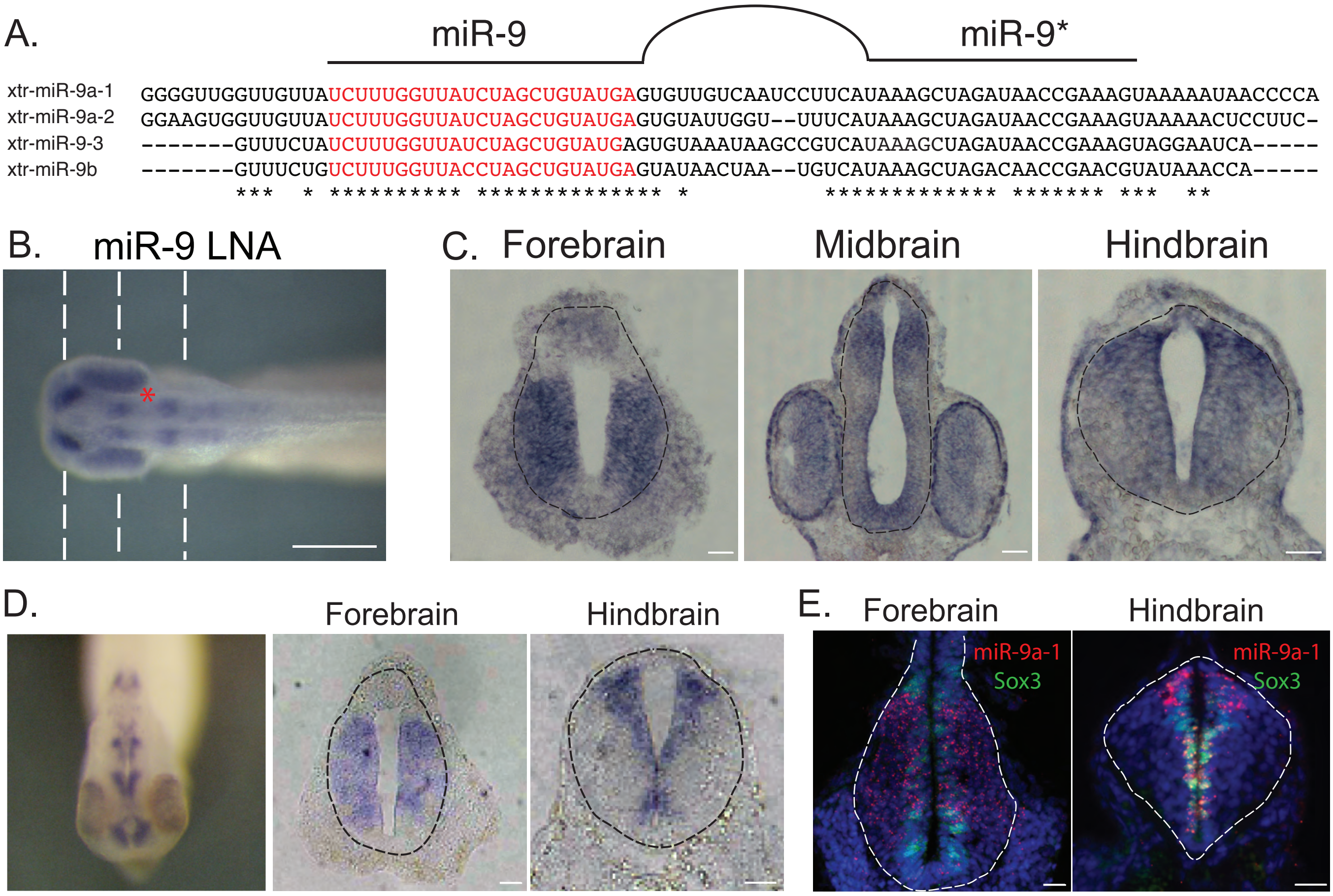

Figure S1. miR-9 expression in *Xenopus tropicalis*

(A) Sequence alignment of miR-9 predicted precursors. Positions that have an identical single residue are marked with an asterisk. Mature miR-9 sequence is shown in red.

(B) Whole mount *in situ* hybridization for mature miR-9 (LNA-based probe). Scale bar = 200 μm

(C) Transverse sections of embryos stained for mature miR-9. Scale bar = 20 μm

(D) *In situ* hybridization for miR-9a-1 primary transcript in stage 36 *X.tropicalis* embryos in whole mount and transverse sections. Scale bar = 20 μm

(E) Fluorescent in situ hybridization (FISH) for miR-9a-1 (in red) combined with immunohistochemistry for Sox3 (marker for neural progenitors) in st. 36 embryo. Scale bar = 20 μm

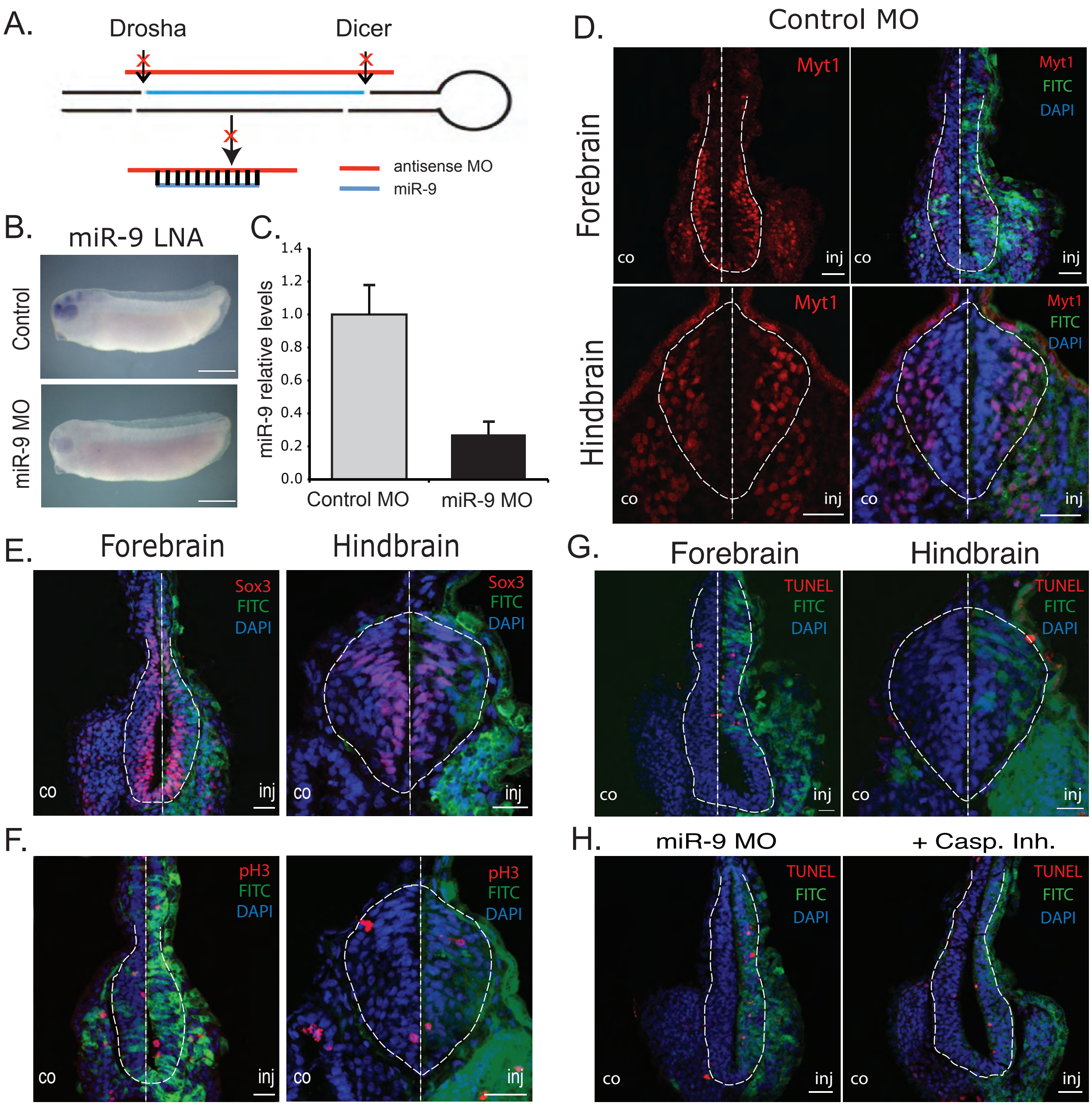

Figure S2. Injection of control morpholino has no effect on neural development

(A) Representative schematic of the mechanism of action of miR-9 MO.

(B) In situ hybridization for mature miR-9 in control and miR-9 MO injected embryos, Scale bars = 200µm

(C) Real-time PCR for mature miR-9 in control MO and miR-9 MO injected embryos, n=3 (10 embryos each), error bars represent SEM

Injection of control MO has no effect on the number of Myt1 positive cells (D), Sox3 positive area (E), phosphohistone H3 positive cells (F) or apoptosis (G).

(H) Injection of caspase inhibitor together with miR-9 MO prevents the apoptosis in the fore-brain as indicated by TUNEL staining.

In all images FITC was used to identify the injected side and DAPI to counterstain the DNA. Neural tube is outlined with a dashed line. Scale bars = 20µm

A.

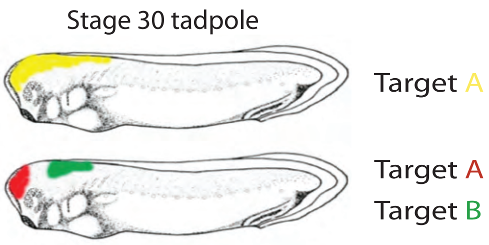

B.

Category	Function	P-value	Molecules
Nervous System Development and F unction	Differentiation of neurons	3.72E-05	BTG2, CNTFR, CNTN4, EFNA1 EN1, EN2, FGF5, GAB2, HES1, ID4, ISL1, KCNMA1, LIFR, LMX1A, NR2E1, NTRK3, PSEN1, REST, RET, RUNX1, SHC1
Cell Cycle	G1/ S phase transition	2.13E-02	ATF3, CCNE1, CCNE2 ELAV1, HES1, MAP2K3, NCOA3, SOX2
Cell Death	cell death	3.06E-03	BTG2, CNTFR, EFNA1, EN1, GAB2, GAB2, HES1, ISL1, KCNMA1, NR2E1, NTRK3, REST, RET, SHC1

C.

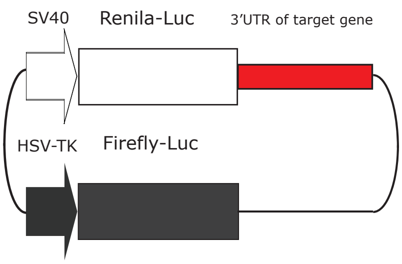

D.

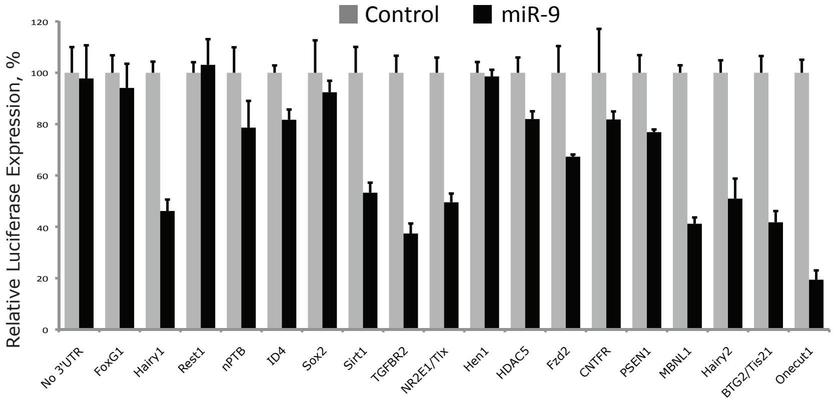

E.

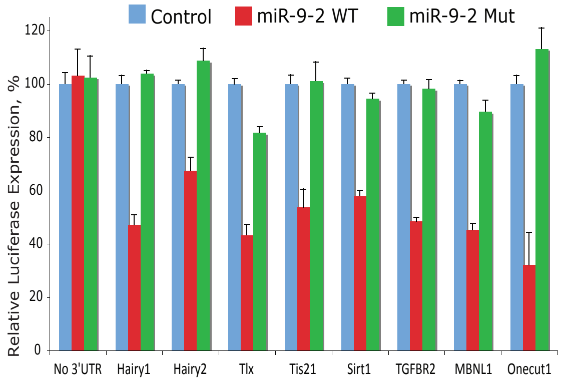

F.

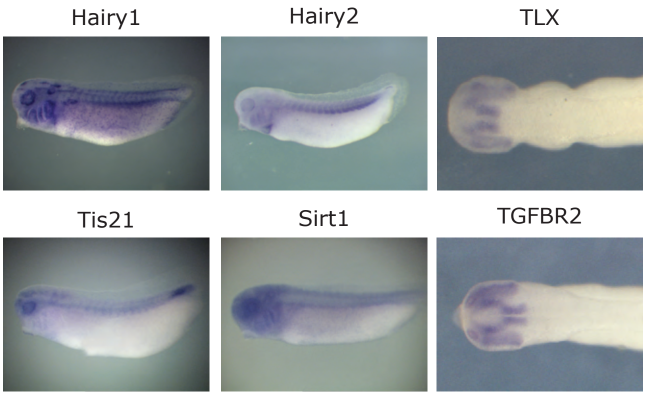

Figure S3. Target screen used to identify several putative miR-9 targets

(A) Possible mechanism of the regional-specific miR-9 MO phenotype: Target A (yellow) is expressed along the A-P axis or Targets A and B are expressed only in the forebrain or hindbrain respectively.
(B) GO Analysis using miR-9 predicted targets in mammals identifies neuronal differentiation, G1/S cell cycle progression and apoptosis among the significant categories. (C) Schematic representation of the psi-CHECK2 luciferase reporter vector used in this study. SV40 and HSV-TK represent promoter regions driving the expression of Renila and Firefly luciferase respectively.
(D) HeLa cells were transfected with luciferase reporter carrying the 3'UTR of the predicted miR-9 target together with scrambled precursors (Control) or miR-9 precursors (miR-9). Luciferase expression was normalized and expressed relative to the control levels. Error bars represent s.d.
(E) A subset of the targets in (D) were further validated using control vector (Control), wild-type (miR-9-2 WT) or mutant (miR-9-2 Mut) miR-9-2. Luciferase expression was normalized and expressed relative to the control levels. Error bars represent s.d.
(F) Whole mount in situ hybridization for hairy1, hairy2, NR2E1/TLX, Tis21/Btg2, Sirt1 or TGFβR2 in Stage 30 X. tropicalis embryos.

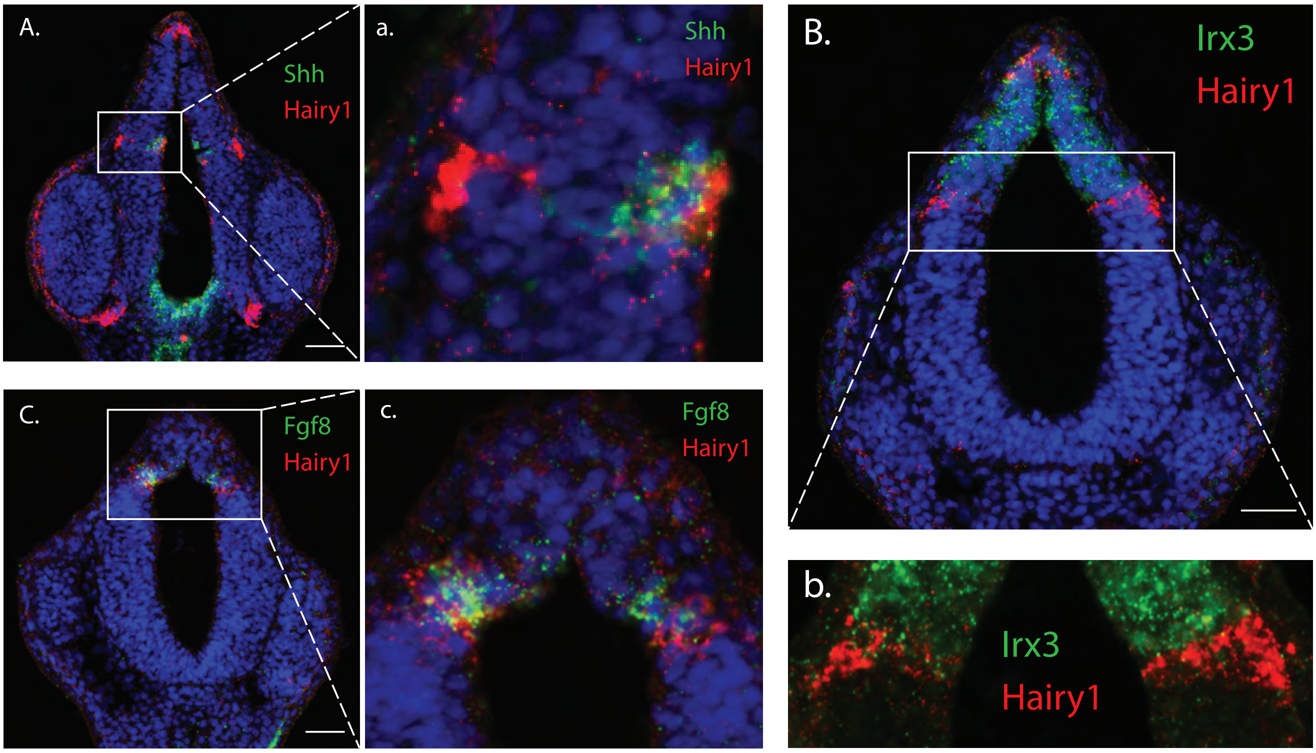

Figure S4. Hairy1 is expressed in the ZLI boundary region and overlaps with Fgf8 domain

(A) *Hairy1* co-localizes with the marker of the ZLI *Shh* in the forebrain region as shown by double fluorescent in situ hybridization. The boxed region is enlarged in (a).

(B) *Hairy1* is expressed immediately adjacent to *Irx3* as shown by double fluorescent in situ hybridization. The boxed region is enlarged in (b).

(C) *Hairy1* expression overlaps with *Fgf8* in the developing dorsal forebrain. The boxed region is enlarged in (c).

In all images DAPI is used to stain the DNA (blue). Scale bars = 20 µm
